# Quantitative Analysis of Carbonation Degree of Mortar Based on Multiple Interactive Influences

**DOI:** 10.3390/ma17235895

**Published:** 2024-12-02

**Authors:** Lihua Xie, Yang He, Zhenghong Tian, Hao Lu, Xinyu Zhang, Lujia Li

**Affiliations:** 1 College of Water Conservancy and Hydropower Engineering, Hohai University, Nanjing 210098, China; 220202050003@hhu.edu.cn (L.X.);; 2 Nanjing Hydraulic Research Institutes, Materials & Structural Engineering Department, Nanjing 210029, China

**Keywords:** multidimensional carbonization, HABT method, carbonization degree, quantitative analysis, interactive influence

## Abstract

This study conducts quantitative analysis of the degree of mortar carbonation under the influence of a multi-dimensional interaction. The HABT method is used to determine the degree of mortar carbonation and is compared with the TGA method. The result shows that the determination result of the HABT method is only 3.86% higher than that of the TGA method. This method is suitable for determining the degree of carbonation. The study analyzes the influence of factors such as water-reducing agents on the degree of carbonation, demonstrates the relationship between pore structure and mortar carbonation, and explores the degree of carbonation of corner areas and general edges. It is found that as time prolongs, the degree of carbonation and carbonation depth will no longer show a linear relationship. Carbonation time also affects the direction of the carbonation front line and the effect of water-reducing agents on concrete carbonation. The degree of carbonation is linearly related to carbonation time and the number of exposed surfaces. The water~cement ratio and the number of exposed surfaces affect the porosity of concrete. There is interaction in the multi-dimensional area of mortar. The degree of carbonation at two-dimensional corners is 1.20~1.25 times that at general edges, and the degree of carbonation at three-dimensional corner areas is 2.03~2.11 times that at general edges. Accounting based on general edges will underestimate the carbon sink capacity of mortar structures.

## 1. Introduction

With the acceleration in industrialization, the emissions of greenhouse gases generated by human activities continue to rise. Carbon dioxide (CO_2_), as the main component [1], has an increasingly significant impact on global warming. To address this challenge, the international community has set reducing carbon emissions as an important goal. China has promised to reach its carbon peak by 2030 and strive to achieve carbon neutrality by 2060 [2]. Therefore, accurately assessing and controlling CO_2_ emissions is particularly important. Cement and concrete are the most widely used building materials in the world after water [3,4,5,6,7]. About one-third of greenhouse gas emissions are generated during their production process [8]. It is expected that the demand for these materials will continue to grow in the coming decades [9]. In fact, the absorption of CO_2_ by concrete cannot be ignored [10,11]. It has significant carbon absorption capacity [11,12,13,14] and can convert CO_2_ into chemically stable carbonates, thereby achieving long-term CO_2_ sequestration [15]. This characteristic helps reverse part of the calcination reaction [16] and significantly reduces the CO_2_ emissions during the life cycle of concrete [5,11,14,17]. If the absorption of CO_2_ by concrete is not considered, the carbon emissions of the cement and concrete industry will be overestimated, which will in turn affect the formulation of relevant policies and the implementation of environmental standards [18]. Thus, the CO_2_ curing of concrete is regarded as a highly potential carbon neutralization technology. One of the key parameters for accurately evaluating its storage capacity is to assess the absorption capacity of CO_2_ [19], and this indicator is manifested as the degree of carbonation of concrete or mortar.

Existing studies have provided important bases for determining the degree of carbonation γ. For instance, Taylor [20] estimated that approximately 24% of CaO is not carbonated, and the degree of carbonation γ is about 0.75. Based on Taylor’s research, Pade and Guimaraes also proposed γ as 0.75 in [14]. Additionally, Takano, H., et al. [13] used a method combining artificially weathered waste concrete and coccolithophore (a type of single-cell marine microalgae) culture. Through experiments, it was concluded that more than 80% of the calcium in concrete is converted into CaCO_3_. Dodoo, A., et al.’s research in [21] indicated that when concrete is broken and exposed for 30 years, about two-thirds of CO_2_ will be reabsorbed. Accordingly, F. Xi proposed in the literature [11] that the average value of γ is 0.8, with a variation range of 0.5 to 1.00.

The above research undoubtedly lays a solid foundation for exploring the degree of carbonation. However, most studies on concrete carbonation are only based on single directional carbonation (1D). In fact, some key parts of concrete structures are simultaneously subjected to carbonation in two mutually perpendicular (2D) or three mutually perpendicular directions (3D) [22]. The steel bars in these parts are affected by the interaction between 2D and 3D carbonation [23], and there is a lack of systematic computational models and multi-dimensional carbonation research. This article is based on the hydrochloric acid back titration (HABT), and constructs a calculation model for the degree of carbonation of mortar γ according to the carbonation mechanism of concrete. It is compared with the TGA analysis method to confirm its efficiency and accuracy. Conducting research on the multiple carbonation of mortar will explore the carbonation characteristics of mortar specimens with different mix proportions, exposed surfaces, carbonation times, and dimensions. Combined with the pore structure and compressive strength, the carbonation degree and durability of mortar corner areas and general edges will be analyzed in depth. This study not only innovates in quantitative analysis methods, but also delves into the impact of multidimensional interactions on mortar carbonation. By proposing new theoretical frameworks and accounting methods, it provides new perspectives and methods for carbonation research.

## 2. Materials and Test Methods

### 2.1. Material

In this study, the ordinary Portland cement used was PO 42.5 grade Portland cement produced by the Huai’an Conch Cement Co., Ltd., Huai’an, Jiangsu, China; it was used to make mortar specimens with a density of 1350 kg/m^3^. The basic physical properties of the cement are shown in Table 1. In order to reduce the influence of the coarse aggregate contour on the carbonation boundary edge line at the mortar corners [24], in this experiment, the fine aggregate is river sand with an average fineness modulus of 2.99, a mud content of 4.0%, and a density of 450 kg/m^3^; the water-reducing agent is HLC-IX polycarboxylic, Nanjing Hydraulic Research Institutes, Nanjing, Jiangsu, China, acid-based high-performance water-reducing agent; tap water with a density of 1000 kg/m^3^. The mix proportions are shown in Table 2. Cement and river sand are invariants, and only the amount of water is changed.

### 2.2. Test Method

#### 2.2.1. Specimen Preparation

We refer to the requirements of Chinese standards “Test method for carbonation of cement mortar (GBT42277-2022)” [25] and the “Test method of cement mortar strength (ISO method) (GB/T 17671-2021)” [26], according to Table 2; we take the corresponding amounts of sand, cement, water, and water-reducing agent for the four mix proportions, pour them into a concrete mixer and stir at a speed of 210 ± 5 r/min for about 3 min. Then, we quickly transfer the well-mixed material into a 40 mm × 40 mm × 160 mm prismatic mold. Next, we place the mold containing the material on a vibrating table and vibrate for 30 s to make mortar specimens. After the specimens are left to stand for 24 h, we remove the molds and place the specimens in a standard curing room with a temperature of 20 ± 2 °C and a relative humidity of 95 ± 5% for curing until they reach the age of 42 days. After reaching that age, we take out the test blocks from the standard curing room 2 days before the test, place them in an electric blast-drying oven, dry them at a constant temperature of 60 °C for 48 h, and then naturally cool them for 48 h.

#### 2.2.2. Carbonation Curing

**Figure 1:**
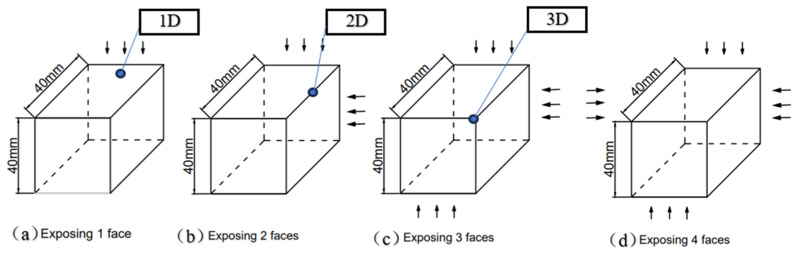
Schematic diagram of exposed surfaces of different mortar specimens.

For the specimens after the drying treatment, we seal the surfaces that do not need to be exposed with paraffin wax that is burned until it is slightly smoky. As shown in Figure 1, the direction indicated by the arrow is the exposed surface. The specimens sealed with wax are transferred into the HTX-X concrete (Suzhou Donghua Experimental Instrument Co., Ltd., Suzhou, Jiangsu, China)carbonation test chamber for accelerated carbonation. We set the CO_2_ concentration to 20 ± 2%, the relative humidity RH 70 ± 5%, and the temperature 20 ± 2 °C. In this test, three specimens with different water~cement ratios form a group, and are exposed on one to four surfaces respectively, as shown in Figure 1. The arrow directions are the exposed directions.

#### 2.2.3. Phenolphthalein Measurement of Carbonation Depth

**Figure 2:**
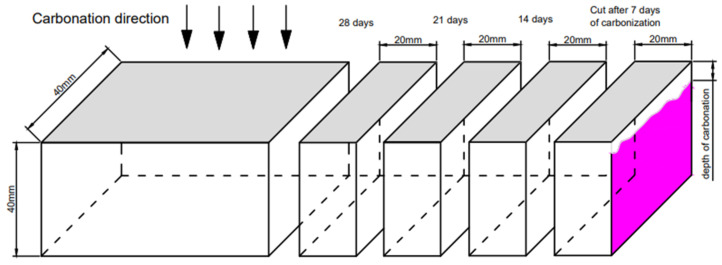
Schematic diagram of mortar carbonation.

**Figure 3:**
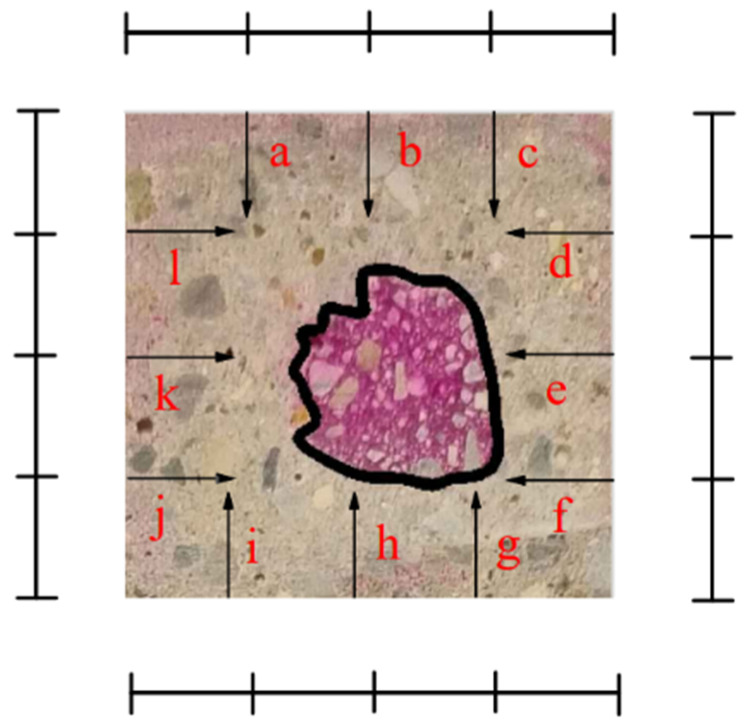
Measurement method of carbonation depth.

Theoretically, the carbonation depth can be determined by the phenolphthalein spray method [19]. Take 1 g of phenolphthalein, dissolve it in 95% ethanol, and dilute it to 100 mL to prepare a phenolphthalein ethanol solution as an indicator. As shown in Figure 2, spray the phenolphthalein indicator on the fresh cut fracture surface of the mortar specimen. The completely carbonated part of the mortar-cutting section retains its original color due to the lack of alkaline substances, while the uncarbonated part will appear purplish-red due to its high alkalinity. After 30 s of color development, use a steel ruler to measure the distance from the measuring points marked every 10 mm on both sides to the color development point, as shown in Figure 3. The letters a to l represent the distance from the line to the boundary before the carbonation of the mortar.Take the arithmetic mean as the average carbonation depth of the mortar.

#### 2.2.4. Preparation of Carbonized Powder

In this study, a multifunctional cutting machine is used for dry cutting, and the blade is cooled before cutting to reduce the impact of high temperature on the carbonation results of mortar specimens. The thickness of each cut is about 2 cm, as shown in Figure 2. The cut slices are stored in sealed bags and wait for representative samples to be selected from the completely carbonated area to be made into powder.

**Figure 4:**
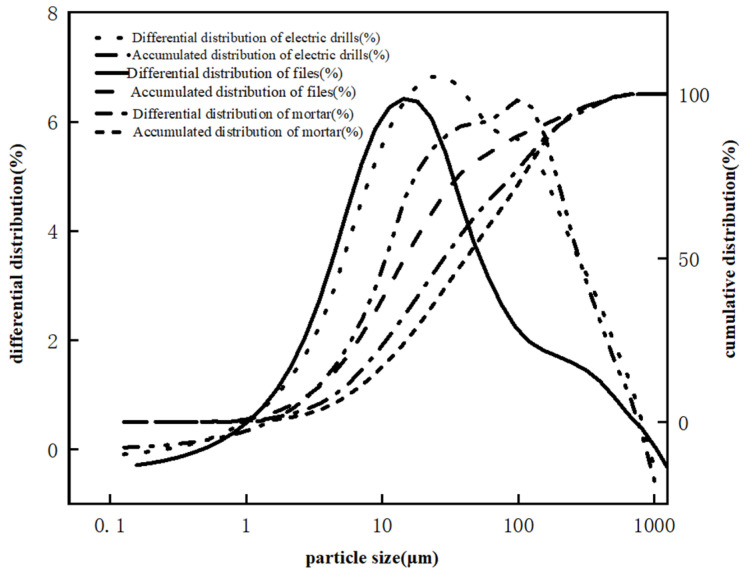
Particle size distribution characteristics of sampling powder.

For mortar specimens, there are many methods for preparing carbonated sample powder. The most common methods are drilling or sawing [24,27,28]. This study compared three preparation methods. The first is to select an appropriate position with an electric drill and to drill an appropriate amount of concrete powder. The second is to use a tungsten steel chisel and a hammer to chisel out part of the mortar sample from the carbonated area of the mortar, and then crush and grind it in a ceramic mortar for about 15 min until it is in powder form. The third is to use a high-temperature quenched fine-toothed file with high hardness and high wear resistance, select an appropriate position, and file the mortar test block to a powder form. The mortar powders obtained by the three sampling methods were analyzed by a laser particle size analyzer (model LS-603). The particle sizes are all greater than 0.45 microns, and the distribution characteristics are shown in Figure 4.

#### 2.2.5. Cleaning and Drying of Carbonized Powder

**Figure 5:**
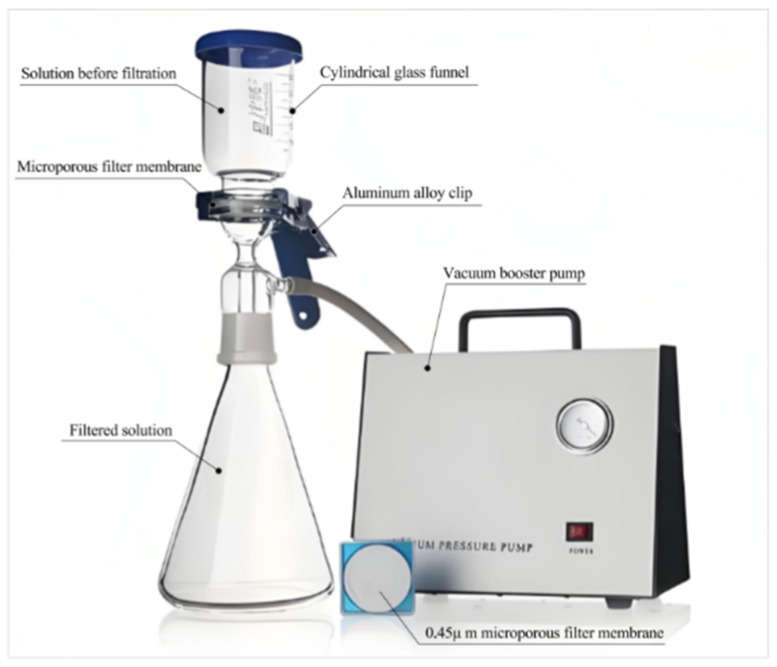
All-glass microporous membrane filter.

To eliminate the interference of Ca(OH)_2_ and others on the experimental results, in this study, an all-glass microporous membrane filter with a filter membrane specification of 0.45 μm as shown in Figure 5 is used to clean and filter the mortar-carbonized powder. First, weigh 0.6 g of concrete carbonized powder with a 10,000th balance and pour it into a cylindrical glass funnel. Next, add 100 mL of distilled water and let the powder soak for 20 min. Then, turn on the vacuum pressure pump for the cleaning operation. During the cleaning process, a total of 2000 mL of distilled water is used to end the cleaning. Finally, collect the solid substances intercepted on the filter membrane and dry them at a temperature of 60 °C for 2 h.

#### 2.2.6. Determination of CaCO_3_ Content in Powder Samples

**Figure 6:**
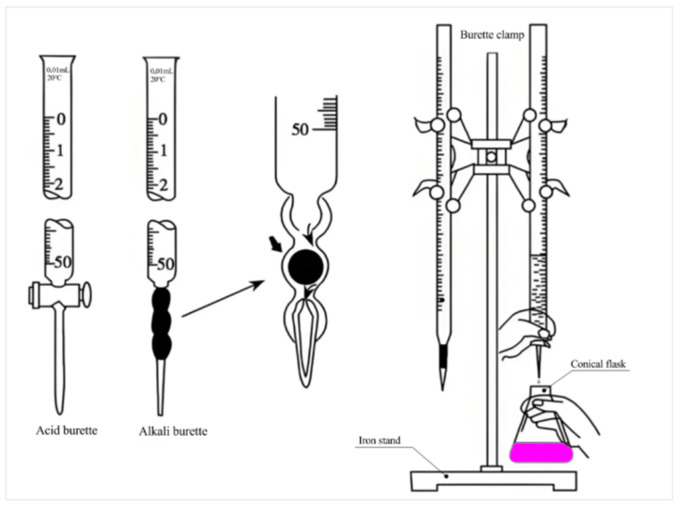
Acid-base burette.

The HABT method is used to determine the content of CaCO_3_ in the powder sample to calculate the degree of carbonation of mortar. The experimental steps are as follows:

(1) Weigh about 0.5 g (m_1_) of the cleaned and dried mortar carbonized powder with a 10,000th balance, accurate to 0.0001 g, and pour it into a 250 mL conical flask as in Figure 6 using standard smooth sulfuric acid weighing paper.
(2) Rinse the wall of the conical flask with a small amount of water to wet the test sample. Slowly add 25.00 mL (v_1_) of hydrochloric acid standard solution with a concentration c(HCl) from the acid burette (in Figure 6), shake the conical flask, cover it with a watch glass, heat and boil and simmer for 2 min. After cooling, rinse the watch glass and flask wall with water, add six or seven drops of phenolphthalein indicator solution, and titrate with v_2_ mL of sodium hydroxide standard titration solution with a concentration c(NaOH) until a faint red color appears. The neutralization reaction proceeds according to the following two equations:

CaCO_3_ + 2HCl = CaCl_2_ + CO_2_ + H_2_O (1)

NaOH + HCl = NaCl+ H_2_O (2)

(3) Use Formula (3) to calculate the mass fraction $W_{{\mathrm{CaCO}}_{3}}$ of the calcium carbonate titration value obtained by the HABT method:

(3) $$W_{{\mathrm{CaCO}}_{3}}=\frac{5.0045\times \left[c\left(\mathrm{HCl}\right)\times V_{1}-c\left(\mathrm{NaOH}\right)\times V_{2}\right]}{m_{4}}$$

In the formula: $W_{{\mathrm{CaCO}}_{3}}$ is the mass fraction of the calcium carbonate titration value, %; c(HCl) is the concentration of the hydrochloric acid standard titration solution, mol/L; c(NaOH) is the concentration of the sodium hydroxide standard titration solution, mol/L; V_1_ is the volume of the hydrochloric acid standard titration solution added, 25 mL; V_2_ is the volume of the sodium hydroxide standard titration solution consumed during titration, mL; m_4_ is the mass of the powder for hydrochloric acid back titration, g; 50.045 is the molar mass of (1/2CaCO_3_), g/mol.

(4) Use Formula (4) to calculate the degree of mortar carbonation γ_1_:

(4) $$\gamma_{1}=\frac{56.08\times W_{{\mathrm{CaCO}}_{3}}\times m_{1}\times m_{5}}{{100\times m}_{2}\times m_{3}\times R}$$

In the formula, m_1_ is the mass of each mortar specimen after carbonation, kg; m_2_ is the cement content in each mortar specimen, kg; m_3_ is the mass of sampling in the mortar carbonation area, g; m_5_ is the mass of powder after cleaning by hydrochloric acid back titration, g.

In summary, the calculation formula for the degree of carbonation γ_1_ by hydrochloric acid back titration method is:

(5) $$\gamma_{1}=\frac{2.8065\times \left[c\left(\mathrm{HCl}\right)\times V_{1}-c\left(\mathrm{NaOH}\right)\times V_{2}\right]\times m_{1}\times m_{5}}{m_{2}\times m_{3}\times m_{4}\times R}$$

Finally, by substituting the obtained γ_1_ into the formula in the literature [11] by F. Xi, the amount of CO_2_ absorbed by the mortar can be obtained. In order to verify whether the data measured by the HABT method are accurate, in this experiment, another identical powder will be analyzed by TGA to compare the differences in the results of the two methods.

#### 2.2.7. TGA Analysis

By using thermogravimetric thermal decomposition to obtain the thermogravimetric curves of mortar specimens in different accelerated carbonation periods, the contents of CaCO_3_ and Ca(OH)_2_ in carbonated specimens can be calculated [12,27,29]. In this study, a TA-TGA-550 thermogravimetric analyzer (TA Instruments Corporation USA, Newcastle, DE, USA)is used, which can simultaneously obtain the thermogravimetric curve (TG) and derivative thermogravimetric curve (DTG) curve of each sample, as shown in Figure 4. Set the temperature range: room temperature—950 °C; heating rate: 20 °C/min; protective gas: N_2_ atmosphere. The following formula is used to calculate the degree of carbonation of the mortar:

(6) $$\gamma_{2}=\frac{{56.08m}_{1}\times m_{6}}{44.01m_{2}\times m_{7}\times R}$$

In the formula, γ_2_ is the proportion of complete conversion of CaO in the mortar by thermogravimetric analysis to CaCO_3_; m_1_ is the mass of each mortar specimen after carbonation, in kilograms; m_2_ is the cement content in each mortar specimen, in kilograms; m_6_ is the weight loss of the powder in the thermogravimetric analyzer between 600 °C and 950 °C, in grams; m_7_ is the mass of the powder weighed for thermogravimetric analysis, in grams; R is the proportion of CaO in cement; 56.08 and 44.01 are the molar masses of CaO and CO_2_, in grams/mol.

When comparing the accuracy of the hydrochloric acid back titration method and the thermogravimetric analysis method, to simplify the analysis process, this study only compares the difference in the value of the degree of carbonation γ.

#### 2.2.8. Preparation and Analysis of Pore Structure Specimens

For the formed mortar test block that has reached the curing age, first, cut it according to the minimum sample number to obtain a cross-section, and try to keep the cutting surface flat and the upper and lower surfaces parallel. The cutting thickness is controlled at about 20 mm. Then, use sandpapers with a fineness of 80, 240, 600, 1000, 1500, and 2000 mesh to polish the observed cutting surface for 15–20 min to make the surface of the test piece cutting surface flat and smooth. Finally, blacken the polished cutting surface with ink. After drying, fill the cutting surface with ultrafine silicon micropowder with a particle size of about 1 μm, and use a scraper to remove the excess powder attached to the surface, so as to obtain an observation surface with a high black-and-white contrast.

**Figure 7:**
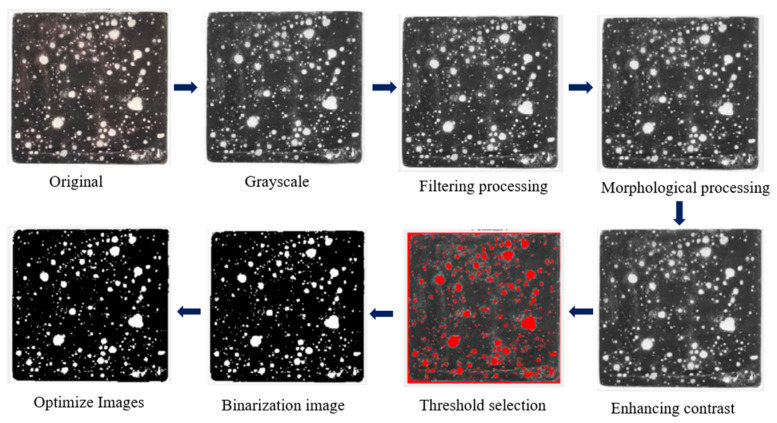
Analysis of mortar-carbonized pore structure by digital image analysis method.

The analysis method used in this study is digital image analysis. It takes pictures of the observation surface by using an imaging system with an IMX766V sensor (Weiwo Mobile Communication Co., Ltd., Dongguan, Guangdong, China) and 50 million pixels to obtain the original RGB image. Subsequently, it is imported into the professional digital image analysis software Baymax 1.3. After steps such as image grayscale processing, image enhancement, image binarization, object segmentation, measurement, and data analysis, the pore structure index of the multi-element mortar-carbonized test piece is finally obtained, as shown in Figure 7.

#### 2.2.9. Compressive Strength Test

In this experiment, the YZH-300·10 type constant (Zhejiang Luda Machinery Instrument Co., Ltd., Shaoxing, Zhejiang, China) loading cement flexural and compressive testing machine with a maximum test force of 300 kN and a constant loading speed of 0.5 kN/s is used to measure the compressive strength of four groups of mortar specimens with different mix proportions after 28 days of carbonation. The specimen size is 40 mm × 40 mm × 80 mm. There are three specimens in one group, and the average value is taken as the final result.

## 3. Results and Discussion

### 3.1. Variation Law of Carbonation Depth

**Figure 8:**
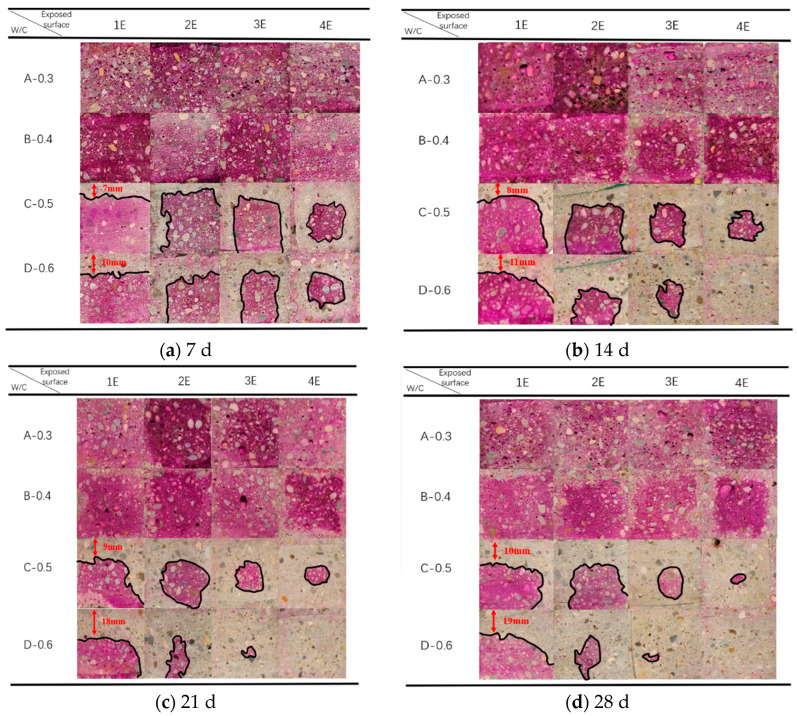
Carbonation depth of mortar with different mix proportions and different ages.

To analyze the variation law of the mortar carbonation depth, four types of mortar with different mix proportions were prepared. Group A and Group B are mortar specimens with water-reducing agent and water–cement ratios of 0.3 and 0.4, respectively. Group C and Group D are mortar specimens with water–cement ratios of 0.5 and 0.6, respectively. The concrete of each mix proportion is placed in four exposure methods, respectively, and subjected to rapid carbonation in a carbon dioxide environment with a concentration of 20%. The carbonation times are 7 days, 14 days, 21 days, and 28 days, respectively. The results are shown in Figure 8a–d. In the figures, from left to right are the carbonation depth changes when exposing one to four surfaces in sequence.

#### 3.1.1. Influence of Water-Reducing Agent on Carbonation Depth

The dosage of the water-reducing agent has a great influence on the carbonation resistance of mortar. Figure 8a,b, respectively, show the variation law of the carbonation depth with the time of mortar mixed with 1.5% and 0.8% polycarboxylate superplasticizer under different exposed surfaces. As can be clearly seen from Figure 8, under the same water–cement ratio, sand ratio and cement dosage conditions, regardless of the carbonation state of several exposed surfaces of Group A and Group B specimens, the Group A specimens with a low water–cement ratio and added water-reducing agent show an obvious purple color during the experiment, and the carbonation depth is basically zero or extremely small. This indicates that the Group A specimens are not carbonated, or the degree of carbonation is extremely low during the experiment. The Group B specimens with the same addition of water-reducing agent also showed no obvious carbonation in the early stage of the experiment. Only in the later stage of carbonation, for example, for Group B 4E starting from 21 days, the carbonation gradually appears at the edge of the exposed surface. The carbonation depth at 28 days is equivalent to the carbonation depth of Group C 1E carbonized for 7 days. These situations fully verify that mortar specimens with a low water–cement ratio have better compactness and carbonation resistance. At the same time, the carbonation time can alleviate the impact of the water-reducing agent on carbonation.

#### 3.1.2. Influence of Water–Cement Ratio on Carbonation Depth

Under the same water–cement ratio and sand ratio conditions, the change in the water–cement ratio also has a great influence on the carbonation resistance of mortar. Figure 8 shows the variation law of the carbonation depth with the carbonation age of mortar specimens with water–cement ratios of 0.3, 0.4, 0.5, and 0.6 on different exposed surfaces. It can be clearly seen that in Figure 8a,b, the carbonation depth of Group A specimens with a water–cement ratio of 0.3 and Group B specimens with a water–cement ratio of 0.4 is extremely small, and only uneven carbonation occurs in 4E at 28 days. In Figure 8c,d, for Group C specimens with a water–cement ratio of 0.5 and Group D specimens with a water–cement ratio of 0.6, the carbonation depth of different exposed surfaces shows the law that the greater the water–cement ratio is, the greater is the carbonation depth. Especially in the early and middle stages of carbonation, for the carbonation of four exposed surfaces, as the water–cement ratio increases in Groups C and D, the carbonation depth increases significantly. However, compared with Group D, the increase in water–cement ratio in Group C has an insignificant impact on the carbonation depth. The reason is that under the same conditions, the greater the water consumption is, the greater is the strength of the mortar, and the porosity of mortar will also increase while the number of large pores will also increase, eventually leading to a decrease in the carbonation resistance of mortar. In addition, it can also be found that in the later stage of carbonation, the influence of the water–cement ratio is significantly increased, which is most prominent in 3E and 4E of Groups C and D.

#### 3.1.3. Influence of Carbonation Time on Carbonation Depth

Figure 8 shows the variation law of the carbonation depth of mortar after carbonation curing for 7 days, 14 days, 21 days, and 28 days with carbonation time. Obviously, the carbonation depth of Group A and Group B with a low water–cement ratio and added water-reducing agent is basically zero or very small during the experiment, but as the carbonation time prolongs, the test specimens are gradually carbonated. The carbonation depth of Group C and Group D specimens with a high water–cement ratio is more sensitive to carbonation time during the experiment. As the time increases, the carbonation depth continuously increases. For example, the carbonation depth of the 1E specimen of Group C gradually increases from 7 mm to 10 mm, and the 1E specimen of Group D increases from 10 mm to 19 mm. This shows that as the carbonation time prolongs, the carbonation process continuously increases [30].

**Figure 9:**
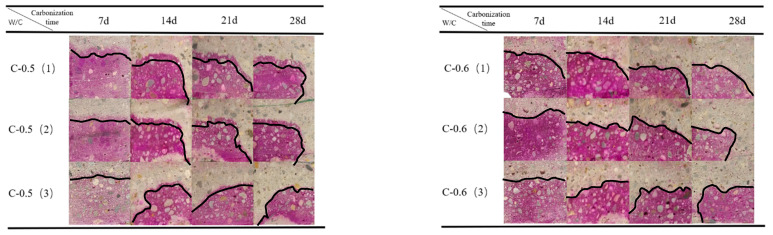
Carbonation depth of three pieces of mortar with the same mix proportion and one exposed surface at different ages.

In addition, it also shows that obvious carbonation also occurs on the unexposed surface. As shown in Figure 8c, the unexposed surfaces of 2E and 3E are clearly carbonated, and the purple area shows a circular gathering phenomenon while the carbonation contour line presents a rounded corner [23], reaching the carbonation effect of 4E. This indicates that the carbonation time will affect the direction of the carbonation front line. To show that this is not an accidental event, we poured three mortar specimens with the same mix proportion and only one exposed surface and at different ages for comparison, as shown in Figure 9. Only on the 7th day of carbonation, the carbonation front lines of the three groups of specimens are relatively flat. As the carbonation time prolongs, their carbonation front lines become more and more curved and then arc-shaped, showing a left or right bending phenomenon. In addition, it can also be found that in the early stage of carbonation, the carbonation area of each group of carbonized specimens is not completely unresponsive to the phenolphthalein reagent, and still reveals an observable purple color. This indicates that there are still alkaline substances in this carbonation area, and that it belongs to an incomplete carbonation area.

It should be particularly pointed out that the carbonation depth of the Group D specimens with a high water–cement ratio is significantly greater than that of other groups, and the longer the carbonation time is, the more obvious its superiority is. For example, the 4E specimen of Group D has been carbonated by nearly half at 7 days, and there is no color development when spraying phenolphthalein at 14 days. This indicates that the specimen has been completely carbonated, and the relationship between the carbonation depth and carbonation time does not satisfy a linear function.

#### 3.1.4. Influence of Exposed Surface on Carbonation Depth

Figure 8 also shows the variation law of the carbonation depth of mortar specimens with the same water–cement ratio and exposed to one, two, three, and four surfaces (1E, 2E, 3E, 4E), respectively, at different carbonation ages. In the early stage of carbonation, the carbonation depth of mortar specimens in Group A and Group B is not greatly affected by the exposed surface. All exposed surfaces of the cutting sections of Group C and Group D show a sharp carbonation front. And as the number of exposed surfaces increases, the carbonation depth of Group C and Group D specimens also gradually deepens. In the later stage, the carbonation of Group A specimens is still not obvious. The 4E of Group B gradually shows carbonation. The specimens of Group C and Group D are severely carbonated. Among them, Group C has been completely carbonated when carbonized for 28 days, and 4E of Group D has been completely carbonated when carbonized for 14 days. In addition, we also observe that the carbonation at the corners is not a theoretical right angle. As marked by the 3E specimen of Group D in Figure 8a and the 2E specimen of Group C in Figure 8d, the carbonation contour line shows a rounded corner. This is the same as Chen’s description in [23], indicating that there is indeed an interaction in the carbonation of multi-dimensional mortar.

### 3.2. Determination of Carbonation Degree by HABT Method and TGA Method

This section discusses the variation law of the degree of mortar carbonation after the CO_2_ entering the sample reacts with cement particles. We mainly use the HABT method to determine the degree of mortar carbonation, and compare the obtained results with the results of the TGA method to judge the accuracy of the HABT method. The samples used in the experiment are the powders taken from the mortar specimens of Group C and Group D with water–cement ratios of 0.5 and 0.6 and exposed on two sides after 28 days of carbonation. The powder components used in the two analysis methods are exactly the same. Among them, the specific numbers of c(HCl), V_2_, c(NaOH), m_1_, m_2_, and R are given below the tables. The experiment is carried out three times, and the average value is taken as the final result. Table 3 is the degree of carbonation measured by the HABT method. The average value of the degree of carbonation of the mortar specimen with a water–-cement ratio of 0.5 measured is 0.2668, and the average value of the degree of carbonation of the mortar with a water–cement ratio of 0.6 is 0.2879.

**Figure 10:**
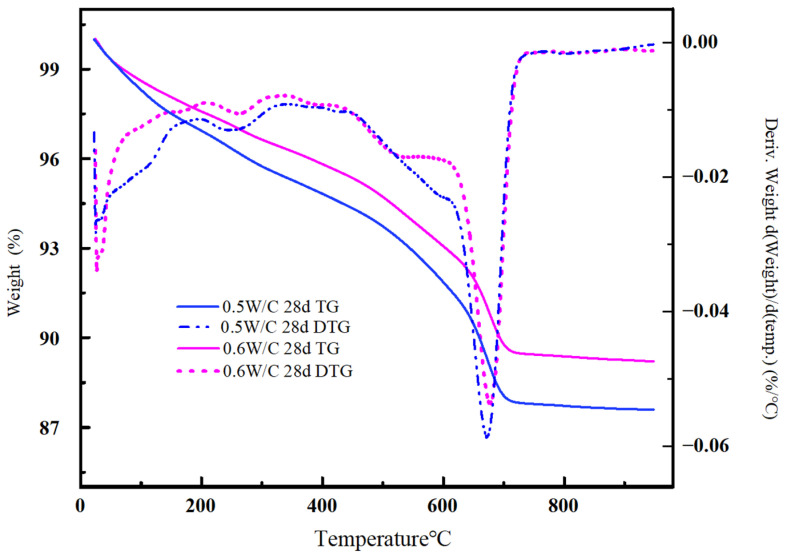
TG and DTG diagrams of concrete with W/C = 0.5 and 0.6 carbonized for 28 days.

Figure 10 shows the TG and DTG spectra of mortar specimens with water–cement ratios of 0.5 and 0.6 after carbonation for 28 days. Both types of concrete powders exhibit sharp weight loss around 680 °C. Overall, the weight loss of mortar specimens with a water–cement ratio of 0.6 is greater than that of specimens with a water–cement ratio of 0.5. Table 4 is the corresponding carbonation degree values obtained by the TGA method, which are 0.2565 and 0.2713, respectively. It can be seen that the results measured by the hydrochloric acid back titration method and the TGA method are slightly different. The former is 3.86% higher than the latter. The main reason may be that some alkaline substances can be cleaned out through Figure 5, but the uncleaned substances can cause excessive use of hydrochloric acid and result in higher measured values; Secondly, the hydrochloric acid titration method covers all free carbon dioxide, resulting in slightly higher results; Finally, there may also be some substances that can react with hydrochloric acid but have not been considered in the TGA method, which may cause the HABT method to produce relatively high values.

According to the carbonation depth of the mortar specimens, the exposed area of concrete, the mix proportion, the degree of carbonation and the amount of CO_2_ absorbed in the carbonation area [12], the amount of CO_2_ absorbed per cubic meter of mortar in concrete structures can be calculated. Taking the average value of the degree of carbonation of the specimen with a water–cement ratio of 0.5 measured above as 0.2668, and substituting the relevant values into the formula in the literature [11] by F. Xi, the amount of CO_2_ absorbed by 1 ton of mortar can be calculated as 37.07 kg. Similarly, the amount of CO_2_ absorbed by 1 ton of mortar e with a water–cement ratio of 0.6 can also be calculated as 51.97 kg. If it is calculated that about 540 kg of CO_2_ is released because limestone is converted into CaO for 1 ton of cement clinker [14,31], the absorbed CO_2_ amounts are equivalent to 7.41% and 10.39% of the CO_2_ emitted during the calcination process, respectively. This proportion does not seem large. The reason may be that the current degree of carbonation is still insufficient. As time goes by, the carbonation process of mortar will continue to be advanced. As shown in Table 5, the calculated degree of carbonation is 51.99%. This result confirms that as estimated by Pade and Guimaraes, 33% to 57% of CO_2_ will be reabsorbed by carbonation [14]. Even in strong carbonation areas, there are still residual clinker particles. It is estimated that there may still be 25% of CaO that may remain uncarbonated [32]. It can be seen that the carbonation degree of mortar is within the range of 50% to 75%. It is necessary to conduct long-term carbonation behavior research to determine whether mortar can be completely carbonized.

### 3.3. Influence of Different Carbonization Time and Exposed Surface on Mortar Carbonation

To further explore the specific effects of different mix proportions, different exposed surfaces, and different carbonation times on the carbonation behavior of mortar, we made Group C specimens with a water–cement ratio of 0.5 and Group D mortar specimens with a water–cement ratio of 0.6. Each group of specimens was set to expose three surfaces and was carbonized for 7 days, 14 days, 21 days, and 28 days, respectively. Subsequently, the HABT method was used to determine the degree of carbonation of the powder in the mortar carbonation zone. The results are presented in Table 5 and Table 6, respectively. Through analysis, it can be clearly seen that the degree of carbonation of Group D with a water–cement ratio of 0.6 is higher than that of Group C with a water–cement ratio of 0.5, with an average increase of about 13%. Moreover, under the same water–cement ratio, the longer the carbonation time of the specimen is, the higher the degree of carbonation will be. The degree of carbonation in the later stage of carbonation of Group C is about 14% higher than that in the early stage of carbonation, and the corresponding increase of Group D is about 18%.

**Figure 11:**
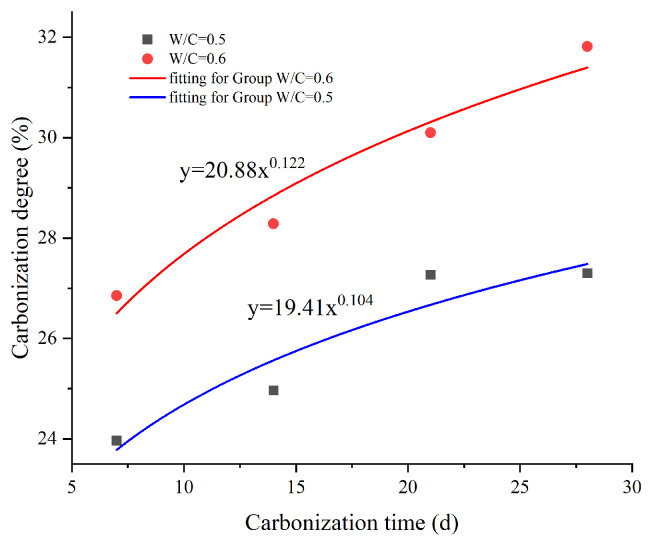
Fitting of carbonation time and carbonation degree of mortar with W/C = 0.5 & 0.6.

**Figure 12:**
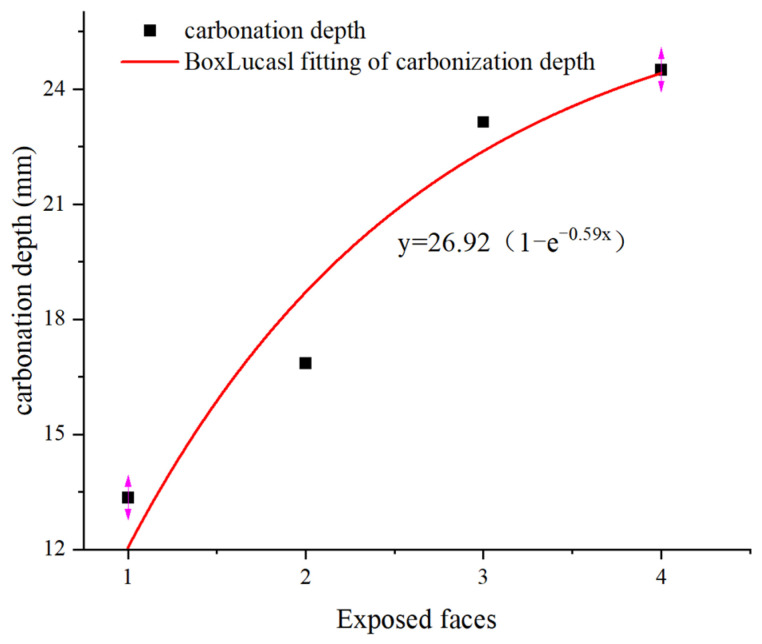
Fitting of exposed surface and carbonation depth of mortar with W/C = 0.5 & 0.6.

To verify the degree of linear correlation between the carbonation time, carbonation depth, and carbonation strength, this study analyzed their correlation. The specific correlation coefficients are shown in Table 7 and Table 8. It can be seen that the correlation coefficients between the carbonation time and carbonation depth and degree of carbonation are all ≥0.8, indicating a high degree of correlation. These fit the experimental results. As shown in Figure 11, the relationship between carbonation degree y_1_ and carbonation time t1 is highly consistent with the Allometric model. The functional relationship is “$y_{1}={a_{1}t_{1}}^{b_{1}}$”. In the same way, this study also measured the degree of carbonation of two groups of specimens with different exposed surfaces under the same mix proportion. The fitting results are shown in Figure 12. The relationship between carbonation depth y_2_ and exposed surface x_2_ is also highly consistent with the Allometric model. The functional relationship is “$y_{2}={26.92(1-e}^{-0.59x_{2}})$”.

### 3.4. Multidimensional Carbonation of Mortar

In Liu’s [18] study, it was assumed that the diffusion depth of CO_2_ on the bottom surface of the mortar specimen is the same as that on the side surface, but the actual experimental results are inconsistent with this assumption. As shown in Figure 8d, for the mortar specimens with three exposed surfaces in Groups C and D, the carbonation depth of each surface is not the same, and a situation similar to that of four exposed surfaces appears, indicating that there is also carbonation on the unexposed surface. In addition, the carbonation contour line in the carbonation area presents a rounded corner [22], indicating an interaction effect. In order to explore the degree of carbonation at the corners and general edges of mortar, the author conducted in-depth research on the degree of carbonation at the corners and general edges of the mortar.

Theoretically, different exposed surfaces are different due to the influence of CO_2_ from different directions. As shown in Figure 8a, it is only affected by CO_2_ from one direction, while Figure 8b,c are affected by CO_2_ from multiple directions. Considering the diffusion of carbon dioxide in three directions, at the corners of the mortar-cutting section, the concentration gradient per unit volume is three times that of the unilateral diffusion. As shown in Figure 1c, the CO_2_ concentration gradient per unit volume at the corner is three times that of the general edge diffusion in Figure 1a.

A set of three mortar specimens were studied and analyzed. The specimens are shown in Figure 1a, 1b, and 1c, respectively. They are exposed to CO_2_ with a concentration of 20% on one, two, and three surfaces, respectively, for accelerated carbonation for 7 days. Figure 1a is one-dimensional carbonation, Figure 1b is a two-dimensional carbonation, and Figure 1c is a three-dimensional carbonation. The degree of carbonation of the mortar-carbonized powder measured by the hydrochloric acid back titration method is shown in Table 9. It can be found that the degree of carbonation of mortar at the two-dimensional corner with a water–cement ratio of 0.6 is 41.44%, which is 1.25 times that of one dimension. The degree of carbonation of mortar at the three-dimensional corner is 51.99%, which is 2.03 times that of one dimension. The degree of carbonation of mortar at the two-dimensional corner with a water–cement ratio of 0.5 is 40.71%, which is 1.20 times that of one dimension. The degree of carbonation of mortar at the three-dimensional corner is 48.85%, which is 2.11 times that of one dimension. This verifies the conclusion that the carbonation degree of the fully carbonated area can exceed 50% [24]. In addition, it also shows that the degree of carbonation of mortar at the corners is affected by the CO_2_ diffused from three adjacent sides and is greater than the degree of unilateral carbonation. This means that if the carbon sink capacity of mortar structures is calculated according to the general edge, the overall value will be greatly underestimated.

### 3.5. Relationship Between Pore Structure and Mortar Carbonation

**Figure 13:**
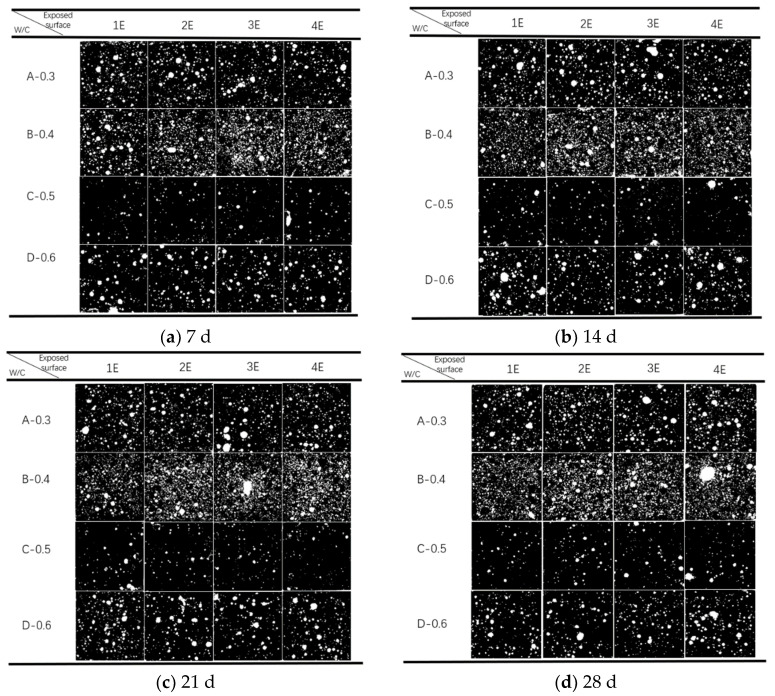
Pore structure of mortar specimens with different proportions, different elements, and different carbonization times.

**Figure 14:**
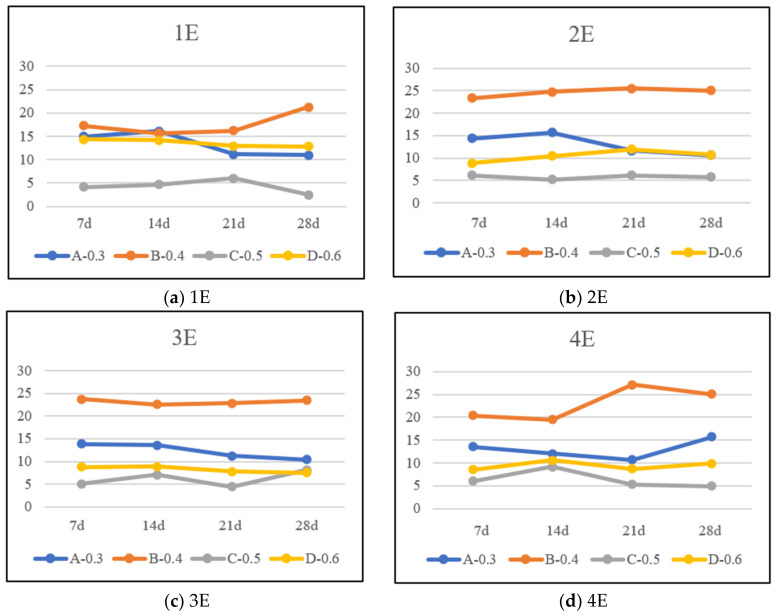
Variation law of porosity of mortar specimens with different mix proportions and different elements carbonized for different times.

Figure 13 shows the porosity structure of mortar specimens after carbonation, and Figure 14 presents the variation law of porosity of mortar specimens with different mix proportions, different carbonation times, and different exposed surfaces. Through observation, it can be found that under the same carbonation time, among the Group A and Group B specimens with water-reducing agent added, the pores of Group B are obviously more dense than those of Group A. This is because in the mortar mix proportion, the more water content there is, the more formed pores will be generated, which in turn leads to an increase in porosity. Correspondingly, for the Group C and Group D specimens without water-reducing agent added, the same rule applies to Group D with a higher water–cement ratio. This fully shows that increasing the water–cement ratio of mortar can improve the water permeability of concrete, and the greater the water–cement ratio is, the greater the porosity of mortar will be and the carbonation depth will also increase. At the same time, it can also be concluded that the specimens without water-reducing agent have fewer pores than the specimens with water-reducing agent. The porosity values in Table 10 strongly support this rule.

In addition, under the same mix proportion, the porosity shows a law of first increasing and then decreasing over time. And the greater the water–cement ratio is and the more exposed the surfaces are, the more obvious this law is. As shown in Figure 13c, the porosity of the Group D specimen exposed on three surfaces begins to increase after 14 days of carbonation; in Figure 13d, the porosity of Group C and Group D exposed on four surfaces gradually increases after 14 days of carbonation. However, specimens with a low water–cement ratio have serious hysteresis. As shown in Figure 13d, specimens of Group A and Group B with a small water–cement ratio only show an upward trend in porosity in the later stage of carbonation. This is consistent with the law shown in Figure 9, that is, they gradually show uneven carbonation in the later stage of carbonation. This further supports the view that carbonation is the cause of changes in the porosity of mortar.

To verify the influencing factors on the porosity of mortar-carbonized specimens, this study carried out a correlation analysis of porosity with carbonation time, carbonation depth, carbonation degree, and exposed surface, as shown in Table 7 and Table 8 specifically. From it, it can be seen that the porosity of the Group C mortar specimens with a low water–cement ratio has a low or weak correlation with the carbonation time, carbonation depth, and carbonation degree, and also has a low or weak correlation with the exposed surface. The porosity of the Group D mortar specimens with a high water–cement ratio has a high correlation with the carbonation time, carbonation depth, and carbonation degree, and has a high or moderate correlation with the exposed surface. This clearly indicates that both the water–cement ratio and the exposed surface are influencing factors on the porosity of mortar carbonation.

### 3.6. Relationship Between Compressive Strength and Mortar Carbonation

**Figure 15:**
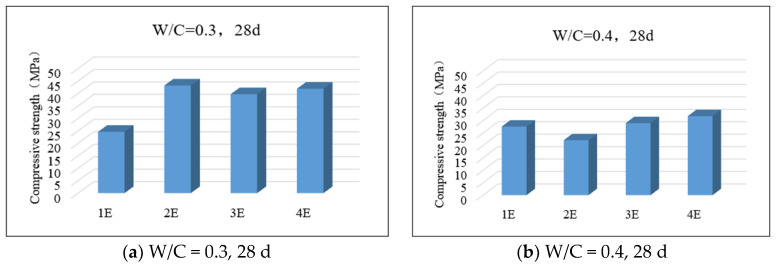

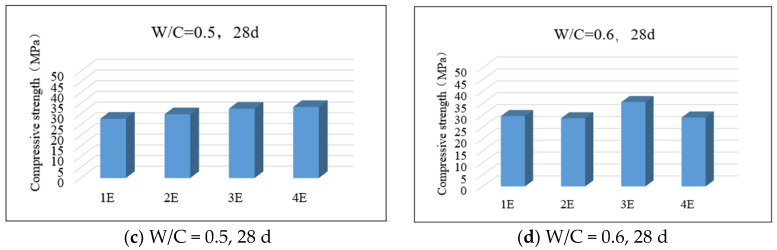
Compressive strength after 28 days of carbonization of mortar with different mix proportions and different exposed surfaces.

Figure 15 presents the compressive strength of mortar specimens with different mix proportions and different exposed surfaces after 28 days of carbonation. As can be seen from the four figures (a), (b), (c), and (d) in the figure, the compressive strength of mortar specimens with a water-reducing agent is significantly higher than that of specimens without a water-reducing agent, indicating that the water-reducing agent can promote the early strength of mortar specimens. For mortar specimens with the same mix proportion, their compressive strength basically increases with the increase in the number of exposed surfaces. When there are fewer exposed surfaces, the mortar specimen with a larger water–cement ratio has a greater compressive strength; however, when the number of exposed surfaces increases, the mortar specimen with a larger water–cement ratio has a lower compressive strength instead. This is because when there are fewer exposed surfaces, the mortar specimen with a larger water–cement ratio has stronger hydration activity and can promote the development of early strength of mortar. When the number of exposed surfaces increases, excessive cement will have more contact with external moisture and air, resulting in a higher degree of carbonation, which in turn affects the compressive strength.

**Figure 16:**
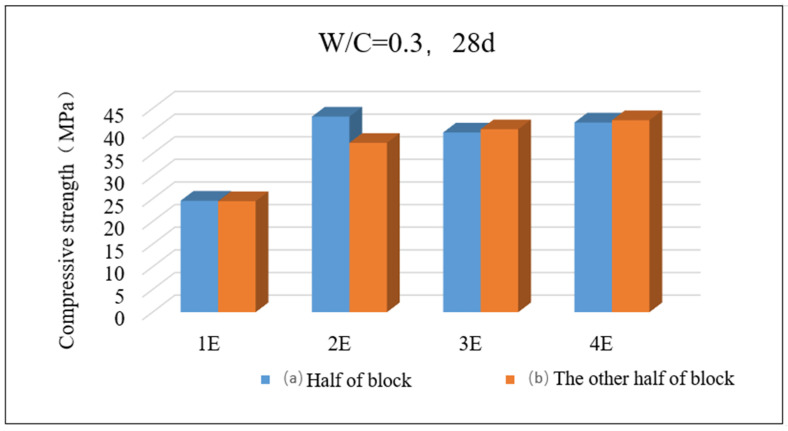
Compressive strength after 28 days of carbonization when W/C = 0.3.

**Figure 17:**
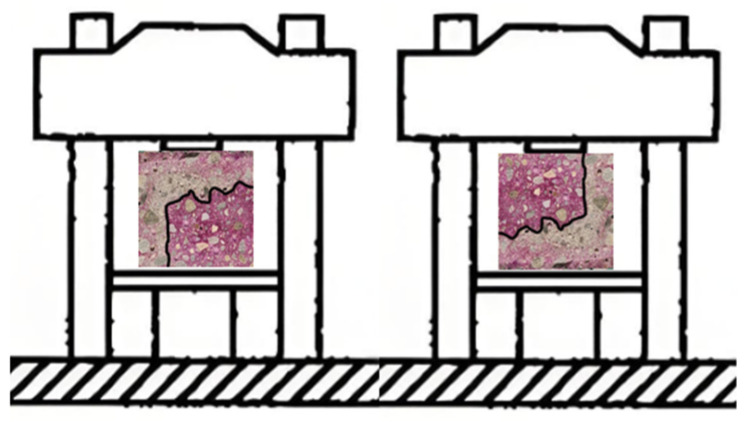
Different placement directions of the same mortar specimen.

It is worth noting that as shown in Figure 16, the compressive strength of the mortar specimen with a water–cement ratio of 0.3 is divided into two parts a and b after 28 days of carbonation. The experiment defaults that the carbonation degree of part a and part b is exactly the same; the specimen size has been changed to 40 mm × 40 mm × 80 mm. As can be seen from the figure, the compressive strengths of parts a and b exposed to one, three, and four surfaces, respectively, are very close, while the compressive strengths of parts a and b exposed to two surfaces are quite different. The reason lies in the fact that there is a difference in the placement direction of these two parts during the experiment, as shown in Figure 17. This situation indicates that an asymmetric carbonization phenomenon like this will cause differences in the compressive strength of the same specimen in different directions. During the compressive strength test, this difference is prone to causing sudden changes in strength. In addition, this study has conducted in-depth analysis on the relationship between the degree of carbonization and compressive strength of mortar specimens. The fitting function “$y_{3}=y_{0}+C\left(\cot h\left(x_{3}-\mathrm{xc}\right)-\frac{1}{x_{3}-\mathrm{xc}}\right)(\cot\mathrm{hz}=\frac{e^{z}+e^{-z}}{e^{z}-e^{-z}}$)” has been successfully obtained, which conforms to the Langevin model.

## 4. Conclusions

This study investigated the carbonation degree of multi-dimensional mortar through theoretical models and experiments, established an accurate and practical model framework, proposed an effective method for determining the carbonation degree of mortar, and summarized the carbonation characteristics of multi-dimensional mortar based on experimental and calculation results. The main conclusions are as follows:

(1) Carbonation time can mitigate the impact of water-reducing agents on mortar carbonation, and this effect is more pronounced on the carbonation depth of mortar specimens with a high water–cement ratio. Generally, a larger water–cement ratio leads to a greater carbonation depth. The functional relationship between the exposed surface and the mortar carbonation depth complies with the Allometric model.
(2) The degree of mortar carbonation is associated with carbonation time, carbonation depth, porosity, water–cement ratio, exposed surface, and compressive strength. Specifically, its functional relationship with carbonation time conforms to the Allometric model, and that with compressive strength adheres to the Langevin model.
(3) There are interactions in multi-dimensional mortar. The carbonation degree at the two-dimensional corner is 1.20–1.25 times that at the normal edge, and in the three-dimensional corner area, it is 2.03–2.11 times that at the normal edge. Calculating based on the normal edge would result in an underestimation of the carbon sink of the mortar structure.
(4) There are slight differences between the results of the mortar carbonation degree measured by the HABT method and the TGA analysis method, with the former being 3.86% higher than the latter. The mortar carbonation degree lies within the range of 50–75%. To determine whether the mortar can be completely carbonated, it is necessary to conduct long-term carbonation behavior research.

## Figures and Tables

**Table 1:** Cement oxide content.

SiO_2_	Al_2_O_3_	Fe_2_O_3_	CaO	MgO	SO_3_	TiO_2_	K_2_O	P_2_O_5_
22.61	5.24	3.82	62.86	0.84	0.40	0.25	0.52	0.10

**Table 2:** Mix proportion of mortar.

Number.	Water~Cement Ratio	Cement	River Sand	Water	Water-Reducing Agent
A	0.3	7.2	21.6	2.16	1.5%
B	0.4	7.2	21.6	2.88	0.8%
C	0.5	7.2	21.6	3.60	-
D	0.6	7.2	21.6	4.32	-

**Table 3:** Determination of carbonation degree γ_1_ by HABT method.

Number	$V_{2}$ mL	$m_{4}$ (g)	$W_{{CaCO}_{3}}$ (%)	$m_{3}$ (g)	$m_{5}$ (g)	γ_1_ (%)	${\bar{\gamma }}_{1}$ (%)
0.5–1	46.18	0.50156	9.75	0.60714	0.52762	26.43	26.68
0.5–2	46.42	0.50206	9.15	0.60728	0.53533	25.16	
0.5–3	46.01	0.50044	10.20	0.60800	0.54401	28.45	
0.6–1	43.94	0.50015	10.19	0.60066	0.52582	27.82	28.79
0.6–2	43.70	0.50064	10.77	0.60012	0.54613	30.57	
0.6–3	44.04	0.50073	9.93	0.60003	0.54185	27.97	

$c\left(HCl\right)$ = 0.4738 mol/L, $V_{2}$ = 25 mL, $c\left(NaOH\right)$ = 0.2480 mol/L, m_1_ = 564.5 g, m_2_ = 160 g, R = 62.86%

**Table 4:** Determination of carbonation degree γ_2_ by TGA method.

Number	m_1_ (g)	m_2_ (g)	m_6_ (g)	m_7_ (g)	R (%)	γ_2_ (%)
0.5	559.4	160	0.2481	6.8556	62.86	25.65
0.6	564.5	160	0.2597	6.8457	62.86	27.13

**Table 5:** Degree of carbonation γ_1_ measured at different carbonization times of Group C.

Number	$V_{2}$ mL	$m_{4}$ (g)	$W_{{CaCO}_{3}}$ (%)	$m_{3}$ (g)	$m_{5}$ (g)	γ_1_ (%)	${\bar{\gamma }}_{1}$ (%)
7d-1	44.52	0.50098	8.75	0.60022	0.54799	25.15	23.96
7d-2	44.64	0.50102	8.46	0.60096	0.54141	23.98	
7d-3	44.81	0.50108	8.04	0.60012	0.53972	22.75	
14d-1	44.39	0.50062	9.08	0.60402	0.53418	25.27	24.97
14d-2	44.12	0.50033	9.75	0.60027	0.52127	26.65	
14d-3	44.61	0.50034	8.54	0.60480	0.51672	22.97	
21d-1	44.2	0.50208	9.52	0.60043	0.54975	27.44	27.27
21d-1	44.11	0.50222	9.74	0.60055	0.52275	26.68	
21d-1	44.14	0.50189	9.67	0.6008	0.54632	27.68	
28d-1	44.16	0.50052	9.65	0.60047	0.54485	27.56	27.30
28d-2	44.16	0.50046	9.65	0.60037	0.53920	27.28	
28d-3	44.24	0.50106	9.44	0.60046	0.54693	27.07	

c(HCl) = 0.4738 mol/L, v_1_ = 25 mL, c(NaOH) = 0.2464 mol/L.

**Table 6:** Degree of carbonation γ_1_ measured at different carbonization times of Group D.

Number	$V_{2}$ mL	$m_{4}$ (g)	$W_{{CaCO}_{3}}$ (%)	$m_{3}$ (g)	$m_{5}$ (g)	γ_1_ (%)	${\bar{\gamma }}_{1}$ (%)
7d-1	43.99	0.50147	9.35	0.60224	0.53147	25.96	26.85
7d-2	44.02	0.50149	9.27	0.60368	0.54451	26.32	
7d-3	43.63	0.50054	10.26	0.60294	0.52816	28.28	
14d-1	43.63	0.5008	10.25	0.60504	0.54795	29.22	28.28
14d-2	43.98	0.5052	9.30	0.6037	0.53706	26.04	
14d-3	43.47	0.50144	10.63	0.60533	0.53501	29.58	
21d-1	43.78	0.50466	9.80	0.60474	0.54188	27.65	30.10
21d-1	43.43	0.50144	10.73	0.60495	0.55138	30.79	
21d-1	43.28	0.50268	11.08	0.60479	0.55254	31.86	
28d-1	43.03	0.50152	11.72	0.60182	0.54452	33.39	31.82
28d-2	43.17	0.50271	11.35	0.60068	0.54288	32.29	
28d-3	43.61	0.50097	10.30	0.60024	0.55145	29.78	

c(HCl) = 0.4738 mol/L, v_1_ = 25 mL, c(NaOH) = 0.2464 mol/L.

**Table 7:** Correlation coefficients of mortar carbonization time, depth, degree, and porosity.

W/C	Element	Carbonization Time (d)	Carbonation Depth (mm)	Carbonation Degree (%)	Porosity (%)
0.5	Carbonization time (d)	1			
	Carbonation depth (mm)	0.97486	1		
	Carbonation degree (%)	0.94697	0.99476	1	
	Porosity (%)	0.49205	0.29059	0.19307	1
0.6	Carbonization time (d)	1			
	Carbonation depth (mm)	0.98829	1		
	Carbonation degree (%)	0.99882	0.99365	1	
	Porosity (%)	−0.92672	−0.97318	−0.94185	1

**Table 8:** Correlation coefficients of mortar-exposed surface, depth, degree, and porosity.

W/C	Element	Exposed Face	Carbonation Depth (mm)	Carbonation Degree (%)	Porosity (%)
0.5	Exposed face	1			
	Carbonation depth (mm)	0.95304	1		
	Carbonation degree (%)	0.62074	0.70609	1	
	Porosity (%)	0.22627	0.30305	−0.38939	1
0.6	Exposed face	1			
	Carbonation depth (mm)	0.98314	1		
	Carbonation degree (%)	0.95017	0.87822	1	
	Porosity (%)	−0.71636	−0.73015	−0.67833	1

**Table 9:** Degree of carbonation at mortar corners and general edges.

	W/C	$V_{2}$ mL	$m_{4}$ (g)	$W_{{CaCO}_{3}}$ (%)	$m_{3}$ (g)	$m_{5}$ (g)	γ_1_ (%)	${\bar{\gamma }}_{1}$ (%)
3D	0.6–1	42.92	0.50065	17.87	0.60852	0.54748	51.05	51.99
	0.6–2	42.72	0.50068	18.37	0.60617	0.5412	52.07	
	0.6–3	42.82	0.50058	18.12	0.60824	0.55873	52.86	
	0.5–1	43.44	0.50057	16.58	0.60732	0.54559	47.06	48.85
	0.5–2	43.14	0.50033	17.34	0.60641	0.54961	49.63	
	0.5–3	43.12	0.50062	17.37	0.60525	0.54977	49.85	
2D	0.6–1	44.85	0.50423	13.70	0.60582	0.54162	38.58	41.44
	0.6–2	44.4	0.50345	14.83	0.60032	0.54252	42.20	
	0.6–3	44.17	0.50942	15.21	0.60202	0.54712	43.54	
	0.5–1	44.59	0.50183	14.41	0.6002	0.53732	40.50	40.71
	0.5–2	44.58	0.50184	14.43	0.6002	0.52982	40.00	
	0.5–3	44.60	0.50165	14.39	0.60025	0.55316	41.63	
1D	0.6–1	43.99	0.50147	9.35	0.60224	0.54946	26.14	25.59
	0.6–2	44.02	0.50149	9.27	0.60368	0.54451	25.64	
	0.6–3	44.01	0.50054	9.31	0.60294	0.52816	25.01	
	0.5–1	44.52	0.50098	8.75	0.60022	0.54799	24.26	23.11
	0.5–2	44.64	0.50102	8.46	0.60096	0.54141	23.13	
	0.5–3	44.81	0.50108	8.04	0.60852	0.54748	21.94	

$c\left(\mathrm{HCl}\right)$ = 0.4972, 0.4972, 0.4738, 0.4738 mol/L, $c\left(HCl\right)$ = 0.4738 mol/L, $V_{1}$ = 25 mL, $c\left(NaOH\right)$ = 0.2480, 0.2464, 0.2480, 0.2464 mol/L, m_1_ = 569.5, 566.5, 564.9, 563.2, 549.8, 544.5 g, m_2_ = 160 g, R = 62.86%.

**Table 10:** Porosity (%) of mortar specimens with different proportions, different elements, and different carbonization times.

Exposed Surface	W/C	7 d	14 d	21 d	28 d
1E	A-0.3	14.98	16.14	11.13	10.96
	B-0.4	17.22	15.67	16.24	21.21
	C-0.5	4.20	4.73	6.00	2.48
	D-0.6	14.30	14.19	12.89	12.82
2E	A-0.3	14.42	15.72	11.68	10.62
	B-0.4	23.34	24.74	25.52	25.04
	C-0.5	6.12	5.22	6.14	5.83
	D-0.6	8.89	10.42	11.97	10.69
3E	A-0.3	13.86	13.61	11.21	10.42
	B-0.4	23.74	22.55	22.77	23.41
	C-0.5	5.06	7.08	4.43	8.14
	D-0.6	8.79	8.85	7.84	7.52
4E	A-0.3	13.58	12.06	10.69	15.69
	B-0.4	20.40	19.46	27.18	25.11
	C-0.5	6.03	9.12	5.26	4.92
	D-0.6	8.53	10.55	8.65	9.82

## Data Availability

The original contributions presented in this study are included in the article. Further inquiries can be directed to the corresponding authors.
